# Incidence and Impact of Routine Inflammatory Parameters on Outcome after Transcatheter Aortic Valve Replacement

**DOI:** 10.1155/2021/6628405

**Published:** 2021-04-15

**Authors:** Polykarpos C Patsalis, Assem Aweimer, Henrik Scharkowski, Dritan Useini, Peter Lukas Haldenwang, Justus Thomas Strauch, Ali Canbay, Andreas Mügge, Antonios Katsounas

**Affiliations:** ^1^Department of Cardiology and Angiology, University Hospital Bergmannsheil, Ruhr University Bochum, Bochum, Germany; ^2^Department of Medicine, Devision of Cardiology and Emergency Medicine, Knappschaft University Hospital, Ruhr University Bochum, Bochum, Germany; ^3^Department of Cardiothoracic Surgery, University Hospital Bergmannsheil, Ruhr University Bochum, Bochum, Germany; ^4^Department of Medicine, Knappschaft University Hospital, Ruhr University Bochum, Bochum, Germany

## Abstract

**Background:**

Previous research reported adverse clinical outcomes in association with systemic inflammation (SI) after transcatheter aortic valve replacement (TAVR). However, data characterizing the impact of SI, as reflected by postprocedural routine inflammatory parameters (pRIP), on clinical outcome of patients undergoing TAVR are sparse.

**Objectives:**

In light of this, the present work aimed to analyze incidence and clinical significance of pRIP after transapical (TA) and transfemoral (TF)-TAVR.

**Methods and Results:**

Data of 81 high-risk consecutive patients undergoing TAVR in our center from 2017 to 2018 were analyzed in a retrospective manner. 40 out of 81 patients (49, 4%) were treated via TF access (group A) and 41 patients via TA access (group B). Incidence, cause, and amplitude of pRIP were analyzed in relation to pre- and peri-interventional data. Assessment of outcomes was conducted according to the valve academic research consortium (VARC-2). Postprocedural C-reactive protein (pCRP) and leucocytes (pL) were significantly increased in patients undergoing TA-TAVR (group B) vs. TF-TAVR (group A; 12.1 ± 9.7 vs. 22.1 ± 7.9 mg/dl, *p* < 0.001 and 12.8 ± 4.0 vs. 14.2 ± 3.8/nl, *p* = 0.002); however, there was no significant difference regarding incidence of postprocedural fever (pF) ≥38.0°C (12.5% vs. 22%, *p* = 0.37). Furthermore, we observed a vast (though insignificant) trend towards a longer fever duration in group B vs. group A (9.9 ± 14.9 vs. 3.2 ± 5.9 hours, *p* = 0.06). Further analysis identified pCRP >30 mg/dl (hazard ratio (HR) 3.15, confidence interval (CI) 1.22–8.14, *p* = 0.018) and European System for Cardiac Operative Risk Evaluation (logistic EuroSCORE I (ES)) >20% (HR 2.95, CI 1.17–7.47, *p* = 0.02) as predictors of mortality; in this context, we also discovered a marginally significant trend for pL > 14/nl (HR 2.44, CI 0.97–6.14, *p* = 0.05). Multivariate analysis by use of the fisher`s exact test revealed a significant association between pCRP >30 mg/dl and ES >20% (*p* < 0.001).

**Conclusion:**

pRIP are significantly increased in patients undergoing TA-TAVR. pCRP >30 mg/dl, ES>20%, and pL > 14/nl are hallmark of adverse prognosis and require further investigation.

## 1. Introduction

Transfemoral transcatheter aortic valve replacement (TF-TAVR) has evolved to the standard of care for patients with severe symptomatic aortic valve stenosis at prohibitive, high, and even intermediate risk for surgical aortic valve replacement [1–4]. Systemic inflammatory response syndrome (SIRS) and fever per se have been associated with worse outcomes after TF-TAVR [5, 6]. Recent research yielded that febrile episodes after TF-TAVR may represent a noninfectious inflammatory response to rupture of heavily calcified plaques during preparatory balloon aortic valvuloplasty (BAV) or valve deployment [6]. However, current data show that direct TAVR without BAV can lead to better outcomes and less adverse events due to the procedural simplification [7]. In addition, performing TF-TAVR with the modern low-profile sheaths enhancing the safety of the procedure can lead to a less traumatic transfemoral device introduction [8].

However, in many cases, alternative approaches for TAVR are necessary due to complicated peripheral vasculature [9]. Data characterizing the impact of postprocedural inflammation on patients' clinical conditions and outcomes after transapical TAVR (TA-TAVR) are sparse. Against this background, the purpose of the present study was to analyze incidence and clinical significance of pRIP after transapical (TA) versus transfemoral (TF)-TAVR.

## 2. Methods

### 2.1. Patient Population

Data from 81 high-risk patients with symptomatic aortic valve stenosis who consecutively underwent transfemoral (group A, *n* = 40) or transapical (group B, *n* = 41) TAVR in our center from 2017 to 2018 using the Medtronic Evolut R (MER) or Medtronic Evolut Pro (MEP), (Medtronic Inc., Minneapolis, MN, USA; *n* = 9), the Edwards SAPIEN 3 (ES3), (Edwards Lifesciences Inc., Irvine, CA, USA; *n* = 62), and the Symetis ACURATE neo (Boston Scientific Corporation, Natick, MA, USA; *n* = 10) bioprosthesis were analyzed.

Incidence, cause, and amplitude of pRIP (leukocytes, C-reactive protein, procalcitonin) in association with definite outcomes were assessed. Peak pRIP up to 72 hours after implantation was evaluated. Decisions for TAVR were met by an interdisciplinary heart team [2, 9, 10]. TAVR procedures were performed according to standard techniques [10–12]. A central venous catheter, an arterial line, and urinary catheterization were used in both groups. Endotracheal intubation was performed in the transapical group. Local anesthesia with conscious sedation was routinely used in the transfemoral group. Patients of the transapical group underwent general anesthesia and were extubated in the intensive care unit (ICU). Patients of the transfemoral group remained in the ICU or intermediate care unit (IMC) for 24 to 48 hours depending on the postinterventional hemodynamic and respiratory status. Direct TAVR was only performed with the balloon expandable bioprosthesis.

This study received ethical approval from the “Ethical Commission of the Ruhr University, Bochum.” Informed consent had been obtained from all individual participants included in the study.

### 2.2. Endpoint

The primary endpoint was all-cause mortality mortality at 30-days and 1-year according to the Valve Academic Research Consortium (VARC II) definitions [12]. Other complications after TAVR with focus on stroke, bleeding, vascular complications, and transcatheter heart valve (THV) performance were recorded and further evaluated [12].

### 2.3. Postinterventional Protocol

After TAVR, patients were admitted for 24 hours to an intensive care unit for postinterventional surveillance. Clinical examination, electrocardiogram, body temperature, and chest X-ray were assessed. All blood parameters, which had been determined by the initial examination, were rechecked. Follow-up visits took place 3 and 12 months after discharge.

### 2.4. Statistics

Categorical data are shown as frequencies and percentages; continuous variables are presented as means and standard deviation. The normal distribution of the variables was verified by the Shapiro–Wilk test (p-Wert ≥ 0.1). Comparisons were performed with 2-sided *χ*^2^-tests or 2-sided Fisher's exact-tests for categorical variables and one-way ANOVA for continuous variables. For multiple testing, Bonferroni correction was additionally used. ANOVA and *t*-test were performed to compare normally distributed variables, and the Mann–Whitney test was used to compare the other nonnormally distributed variables between the 2 groups. A *p* value <0.05 defined significance. Survival analyses were performed by the Kaplan–Meier curve, with patients censored as of the last date known alive. All statistical analyses were performed using SPSS (version 17.0, SPSS, Chicago, IL, USA). The authors had full access to the data and take full responsibility for their integrity. All authors have read and agree to the manuscript as written.

## 3. Results

### 3.1. Baseline and Postprocedural Characteristics

Our study cohort represents a typical TAVI patient population at high risk for open-heart surgery with symptomatic aortic stenosis. The transapical patients (group B) had a significantly higher European System for Cardiac Operative Risk Evaluation Score (logistic EuroSCORE I, 21.9 vs. 15.3%, *p* = 0.02). There were no other significant differences in the baseline postprocedural characteristics between the 2 groups (Table 1). At least, moderate paravalvular leakage (PVL) after TAVR, previously associated with worse outcome [13–15], did not occur at any patient.

### 3.2. PRIP after TAVR

Postprocedural C-reactive protein (pCRP) and postprocedural leucocytes (pL) were significantly higher in the transfemoral group A vs. transapical group B (22.1 ± 7.9 vs. 12.1 ± 9.7 mg/dl, *p* < 0.001 and 14.2 ± 3.8 vs. 12.8 ± 4.0/nl, *p* = 0.002). There were no significant differences regarding postprocedural fever (PF) ≥ 38.0°C (22 vs. 12.5%, *p* = 0.37). There was a tendency regarding longer fever duration in group B (9.9 ± 14.9 vs. 3.2 ± 5.9, *p* = 0.06). None of the patients had positive blood culture read-outs. Only 3 patients had positive procalcitonin assays (a level≥0.5 ng/mL is considered to be positive, Roche Diagnostics).

### 3.3. PRIP after TAVR and Associated Mortality

Further analysis suggested pCRP >30 mg/dl (hazard ratio (HR) 3.15, confidence interval (CI) 1.22–8.14, *p* = 0.018), and logistic EuroSCORE I (ES) > 20% (HR 2.95, CI 1.17–7.47, *p* = 0.02) as predictors of mortality. There was an almost significant trend regarding pL > 14/nl (HR 2.44, CI 0.97–6.14, *p* = 0.05). Multivariate analysis by use of fisher`s exact test revealed a significant association between pCRP >30 mg/dl and logistic EuroSCORE I >20% (*p* < 0.001).

**Figure 1:**
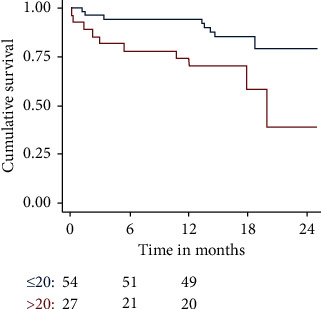
Cumulative survival of patients with Logistic EuroSCORE I (ES) > 20 vs. ≤ 20% patients with ES > 20% had a worse outcome. ES > 20% was a significant predictor of mortality (*p* = 0.02).

**Figure 2:**
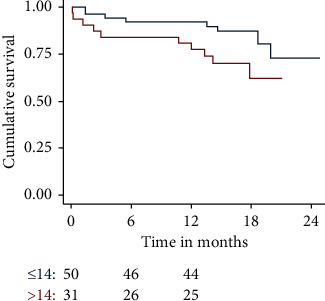
Cumulative survival of patients with postprocedural leucocytes (pL) > 14 vs. ≤ 14/nl. Further analysis showed a statistically nonsignificant trend towards a better outcome for patients with pL ≤ 14/nl (*p* = 0.05).

**Figure 3:**
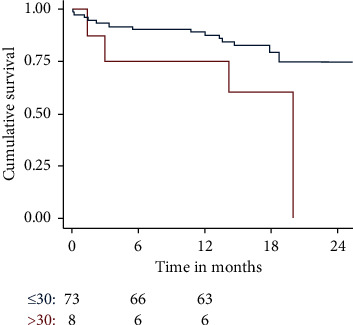
Cumulative survival of patients with postprocedural c-reactive protein (pCRP) > 30 vs. ≤ 30 mg/dl. Patients with pCRP >30 mg/dl had a worse outcome. PCRP >30 mg/dl was a significant predictor of mortality (*p* = 0.018).

Survival at 30 days and 360 days was worse in patients with logistic EuroSCORE I > 20% (*n* = 27), pL > 14/nl (*n* = 31), and pCRP >30 mg/dl (*n* = 8) (Figure 1–3).

## 4. Discussion

The present study demonstrates that SI, as reflected by pRIP, is associated with worse clinical outcomes after TAVR. This analysis is the first to propose a feasible postprocedural inflammatory algorithm to predict mortality after TA- and TF-TAVR in association with the EuroSCORE. These data also indicate that the transfemoral approach for TAVR and associated procedural simplification can lead to lower prevalence of pRIP and, thus, favorably influence attenuation/prevention of systemic inflammatory response syndrome (SIRS). Procedural simplification could also explain lower fever duration after TF-TAVR compared to TA-TAVR, which has been previously associated with increased mortality [5, 6].

### 4.1. Inflammation after TAVR and Associated Mortality

In this study, pRIP after TAVR was not attributed to an infectious origin. Recent studies have shown that fever after TAVR is common and most likely represents a noninfectious postprocedural SIRS [5, 6]. Mechanisms of tissue injury resulting in immunological changes may explain this inflammatory development [5]. SIRS after TAVR may represent a response to rupture of heavily calcified plaques during BAV or valve deployment [6]. In addition, tissue injury occurs not only during introduction of a transfemoral sheeth for TF-TAVR but also during surgical preparation of the apex for performing TA-TAVR.

Peri- and postprocedural cytokine release could be a possible reason for development of a nonspecific inflammation after TAVR. The cytokine-related pathway of SIRS leading to hypotension and not adequate organ perfusion could be also found during many steps of the TAVR procedure [5, 6]. Rapid pacing for implantation or pre- and postdilatation, possible microtraumatic annular injuries during implantation, or tissue damage of the peripheral vasculature of the apex of the heart caused by the introduction of the sheeth can create a SIRS-like constellation. Even preforming TAVR without complication seems to be able to still trigger systemic inflammatory response; however, this postulation lacks robust evidence. Interestingly, none of the patients in this analysis had positive blood culture read-outs, and only 3 had increased serum-procalcitonine levels.

The results of this analysis present a simple, easily available algorithm in order to recognize clinically relevant postprocedural inflammation, which may influence the clinical outcome. Incidence of the suggested postinterventional PCRP and PL cut-off values in patients with increased logistic EuroSCORE I> 20% appears to carry adverse prognosis, and patients with such combination should better remain under more intensive postinterventional surveillance. In addition, it is of great importance to break this suspected vicious cytokine-circle before critical inflammation occurs. Therefore, prospective studies investigating broader inflammation parameters are necessary in order to evaluate if procedure simplification (e.g., modern direct TAVR without preparatory BAV and use of a selfexpandable bioprosthesis for avoiding rapid pacing) can play a role, especially for the very elderly and markedly vulnerable high-risk population.

### 4.2. Impact of Procedure Simplification during TAVR on Reduction of Associated Complications

Current data show that direct TAVR, performed without the use of preparatory BAV, can simplify the procedure and consequently lead to lower complication rates [7]. Direct TF-TAVR helps interventionists to reduce exchange maneuvers in the aortic arch and the left ventricle and renders additional rapid pacing (that is needed for the preparatory BAV) unnecessary. These recent data show that procedure simplification, in general, can lead to favorable outcomes [7, 8]. In addition, the impact of the current low-profile sheaths (14–16 Fr) offer a gentler, atraumatic sheath introduction over the transfemoral access route [8]. The transapical approach is per se more invasive and traumatic. In addition, patients undergoing TA-TAVR are nowadays not suitable for the transfemoral approach and commonly at higher operative risk. This could partly explain the lower incidence of pRIP after TF-TAVR in the current analysis. In line with this, there was a tendency regarding longer fever duration in the transapical group. Local anesthesia with conscious sedation routinely used for the transfemoral group could have had a positive impact regarding lower incidence of postprocedural inflammation; nevertheless, this remains hypothetical.

## 5. Limitations

Our data are derived from a retrospective analysis of consecutive patients and not from a prospective, randomized trial. Patients undergoing TA-TAVR were not suitable for the transfemoral access route and, therefore, due to complicated peripheral vasculature possibly at higher operative risk. Further prospective investigation is necessary to evaluate if direct implantation and associated procedure simplification, in general, could have a positive impact in reducing the extent of postinterventional inflammation.

Of note, the significant association between pRIP and EuroSCORE raises the question, whether patients at higher operative risk are more likely to develop SIRS due to increased fragility or incidence of SIRS remains random but has a higher impact on outcome if very vulnerable patients are affected. This correlation needs further investigation. In addition, possible negative impact of the use of general anesthesia and associated prolonged recovery in the ICU requires further research.

## 6. Conclusions

Inflammation after TAVR is common and most likely in terms of a SIRS. PRIP are significantly higher in patients undergoing TA-TAVR. PCRP >30 mg/dl, logistic EuroSCORE I >20%, and pL > 14/nl carry adverse prognosis and require further investigation.

## Figures and Tables

**Table 1:** Baseline and postprocedural characteristics.

A	Overall (*n* = 81)	TF-TAVR (*n* = 40)	TA-TAVR (*n* = 41)	*p* value
Age, years	82.1 ± 6.0	82.9 ± 4.9	81.2 ± 6.9	0.5
Male gender	40 (49.4)	19 (47.5)	21 (51.2)	0.8
Weight, kg	77.3 ± 16.5	74.6 ± 14.7	80.0 ± 17.9	0.1
Height, cm	167.8 ± 9.1	168.3 ± 9.1	167.2 ± 9.2	0.5
Logistic Euroscore I (%)	18.6 ± 12.7	15.3 ± 9.5	21.9 ± 14.6	0.02
Aortic valve area, cm2	0.8 ± 0.3	0.8 ± 0.3	0.8 ± 0.3	0.7
Mean transvalvular PG before TAVR, mmHg	38.5 ± 14.7	38.8 ± 12.7	38.0 ± 17.4	0.8
LVEF (%)	52.9 ± 13.7	56.8 ± 9.9	49.0 ± 15.8	0.05
CAD	41(50.6)	22 (55)	19 (46.3)	0.5
Prior MI	12 (14.8)	5 (12.5)	7 (17.1)	0.7
Prior PCI	28 (34.6)	15 (37.5)	13 (31.7)	0.6
Prior heart surgery	12 (14.8)	3 (7.5)	9 (22)	0.1

B				
Mean transvalvular PG after TAVR, mmHg	9.3 ± 4.0	9.2 ± 4.2	9.5 ± 3.7	0.5
Vascular complications (major)	1 (1.2)	1 (2.5)	0 (0)	0.4
Vascular complications (minor)	2 (2.5)	2 (5)	0 (0)	0.2
Stroke (disabling)	0 (0)	0 (0)	0 (0)	—
Stroke (nondisabling)	2 (2.5)	2 (5)	0 (0)	0.2

Values are mean ± SD, *n* (%). CAD = coronary artery disease, LVEF = left ventricular ejection fraction, MI = myocardial infarction, PCI = percutaneous coronary intervention, PVD = peripheral vascular disease, PG = pressure.

## Data Availability

The survey data used to support the findings of this study are available from the corresponding author upon reasonable request.

## Abbreviations

PL: Postprocedural leucocytes
pCRP: Postprocedural C-reactive protein
PRIP: Postprocedural routine inflammatory parameters
PVL: Paravalvular leakage
TA: Transapical
TAVR: Transcatheter aortic valve replacement
TF: Transfemoral.
